# Effects of Fogging System and Nitric Oxide on Growth and Yield of ‘Naomi’ Mango Trees Exposed to Frost Stress

**DOI:** 10.3390/life13061359

**Published:** 2023-06-09

**Authors:** Hosny F. Abdel-Aziz, Ashraf E. Hamdy, Ahmed Sharaf, Abd El-wahed N. Abd El-wahed, Ibrahim A. Elnaggar, Mahmoud F. Seleiman, Magdy Omar, Adel M. Al-Saif, Muhammad Adnan Shahid, Mohamed Sharaf

**Affiliations:** 1 Department of Horticulture, Faculty of Agriculture, Al-Azhar University, Cairo 11884, Egypt; 2 Soils and Water Department, Faculty of Agriculture, Al-Azhar University, Cairo 11884, Egypt; 3 Plant Production Department, College of Food and Agriculture Sciences, King Saud University, P.O. Box 2460, Riyadh 11451, Saudi Arabia; 4 Department of Agriculture Botany, Faculty of Agriculture, Al-Azhar University, Nasr City, Cairo 11651, Egypt; 5 Horticultural Science Department, North Florida Research and Education Center, University of Florida/IFAS, Quincy, FL 32351, USA; 6 Department of Biochemistry, Faculty of Agriculture, AL-Azhar University, Nasr City, Cairo 11651, Egypt; 7 Department of Biochemistry and Molecular Biology, College of Marine Life Sciences, Ocean University of China, Qingdao 266003, China

**Keywords:** *Mangifera indica* L., chilling stress, protection, survival, fruit quality, nitric oxide, enzyme activities

## Abstract

In years with unfavorable weather, winter frost during the blossoming season can play a significant role in reducing fruit yield and impacting the profitability of cultivation. The mango Naomi cultivar *Mangifera indica* L. has a low canopy that is severely affected by the effects of frost stress. As a result of the canopy being exposed to physiological problems, vegetative development is significantly inhibited. The current investigation aimed to study the influence of spraying nitric oxide and fogging spray systems on Naomi mango trees grafted on ‘Succary’ rootstock under frost stress conditions. The treatments were as follows: nitric oxide (NO) 50 and 100 μM, fogging spray system, and control. In comparison to the control, the use of nitric oxide and a fogging system significantly improved the leaf area, photosynthesis pigments of the leaf, the membrane stability index, yield, and physical and chemical characteristics of the Naomi mango cultivar. For instance, the application of 50 μM NO, 100 μM NO, and the fogging spray system resulted in an increase in yield by 41.32, 106.12, and 121.43% during the 2020 season, and by 39.37, 101.30, and 124.68% during the 2021 season compared to the control, respectively. The fogging spray system and highest level of NO decreased electrolyte leakage, proline content, total phenolic content, catalase (CAT), peroxidases (POX), and polyphenol oxidase (PPO) enzyme activities in leaves. Furthermore, the number of damaged leaves per shoot was significantly reduced after the application of fogging spray systems and nitric oxide in comparison to the control. Regarding vegetative growth, our results indicated that the fogging spray system and spraying nitric oxide at 100 μM enhanced the leaf surface area compared to the control and other treatments. A similar trend was noticed regarding yield and fruit quality, whereas the best values were obtained when the fogging spray system using nitric oxide was sprayed at a concentration of 100 μM. The application of fogging spray systems and nitric oxide can improve the production and fruit quality of Naomi mango trees by reducing the effects of adverse frost stress conditions.

## 1. Introduction

Mango (*Mangifera indica* L.) is a significant climacteric tropical fruit that is frequently harvested at the full, hard, green pre-climacteric stage [1]. It has a high export potential due to its enticing flavor, scent, and nutritional value [2]. In some regions, mango trees can suffer from chilling stress throughout the winter, which interferes with their typical growth and development [3]. Tropical trees such as mango require protection against chilling damage since they are extremely sensitive to low temperatures [4]. Even in subtropical and tropical climate zones, cold fronts throughout the winter can cause exceptionally low temperatures, especially at night, which frequently can cause effects on agriculture productivity [5]. Data from the Central Laboratory for Agricultural Climate (CLAC) show that throughout the past 10 years, minimum temperatures during the winter season were varied [6]. Mango thrives in a warm, frost-free climate with a designated dry winter season [7]. Mango’s ideal temperature is between 24 and 26.7 °C, with a minimum temperature of 10 to 12 °C, and signs of chilling injury appear below these ranges [3]. 

Low temperatures near the ground are brought on by solar energy radiating into the upper layers of the atmosphere at night [8]. Chilling stress changes a number of cytological, biochemical, physiological, and molecular processes [9]. They include of antioxidant activities, photosynthesis, plasma membrane permeability, hydration status, osmotic balance, and other processes [10]. Cold temperature (0–12 °C) stress can limit the production and geographical dispersion of many economically significant plant species [11]. Tropical and subtropical tree species, such as mango, are exposed to cold temperatures (below 10 °C) [12] on an annual basis in tropical and subtropical latitudes, which can cause significant injury to mango trees [13,14]. Fluctuating temperatures during mango flowering can negatively affect pollen grain germination and fruit formation [15]. Frost is the most serious threat to mango tree survival [16]. Thus, mango is best produced in frost-free locations or in places with periodic light frosts [17]. Immature mango trees can be severely harmed if the temperature falls below −1.11 °C; however, older trees can endure temperatures as low as −3.33 °C for brief periods of time [18]. Furthermore, low temperatures can be harmful to plants [19]; however, protective systems can mitigate the consequences of such stress [20].

Sprinklers utilize the energy released as water freezes and transforms from a liquid to a solid to maintain the temperature of the ice at 0 °C, preventing freezing injuries. The temperature of the ice will remain at 0 °C as long as you keep it moist. The ice will become colder than the ambient temperature if it dries out and water begins to evaporate from it. Citrus trees should be sprinkled with water throughout the frost. As the water freezes around the leaves and branches, it will liberate enough heat to maintain the tissue temperature at 0 °C. This strategy will only be successful if the water is flowing continuously throughout the frost and if it remains flowing until the air temperature is above 2.80 °C [21]. Some growers of apples and grapes also employ this approach. Sprinklers are quite useful in some situations, but when utilized improperly, they can potentially cause injury. Only the leaves that are sprinkled will not suffer freeze damage, although there could be some limb breakage due to accumulation of ice [22].

Nitric oxide (NO) is a redox, gaseous, highly reactive nitrogen species (RNS) generated in living cells under normal and stressful situations [23]. When a plant’s ROS concentration becomes hazardous, nitric oxide may operate as a detoxifier, minimizing any negative consequences [24]. NO can serve as a bioactive molecule that plays an important role in many key physiological processes in plants [25], such as seed germination and dormancy, growth and development of plant tissue, plant cell maturation and senescence, flowering, hormone responses, stomatal closure, and programmed cell death [26]. Furthermore, NO is suggested to be involved in regulating the multiple plant responses to a variety of abiotic and biotic stresses, including drought, salinity [27], extreme temperature, UV radiation, mechanical injury, herbicide, heavy metal, and pathogen attack [28]. NO is itself a reactive nitrogen species and its effects on different types of cells have proved to be either cytoprotective or cytotoxic, depending on both the concentration of NO and the situation of the tissue where it is acting [29]. It can act as an antioxidant able to scavenge ROS to protect plant cells from oxidative damage. Several studies have indicated that the protective effect of NO against abiotic stress is closely related to the NO-mediated reduction in ROS in plants [30]. Wang et al. [31] demonstrated that exogenous NO was an effective option to reduce chilling stress (CI) by inducing the expression, activation, or eliciting of antioxidant enzyme activities in banana fruit. Thus, it appears that the ability of NO to reduce the occurrence of CI is correlated with enhanced antioxidant enzyme activity, an increase which contributes to the adaptation of plants to cold stress and ameliorates oxidative damage such as lipid peroxidation and H_2_O_2_ contents [32]. Moreover, NO treatments induced higher activities of enzymes than those observed in the control during storage of Rish Baba cv. grape [33]. Cold exposure caused transitory phosphorylation of sphingolipids in this environment, and NO was discovered to be a negative regulator of this sphingolipid phosphorylation [34]. A hovering fog can be manufactured and dispersed across the orchard to provide an artificial insulating layer and a water–ice coating. However, limited studies are available regarding overcoming the frost injury of mango trees with foliar application of NO and fogging irrigation systems.

Therefore, the present study aims to study the effects of the foliar application of NO and a fogging irrigation system to overcome frost injury and improve productivity and fruit quality of Naomi mango trees grown under cold stress conditions during the growing season.

## 2. Materials and Methods

### 2.1. Experimental Site

In a private orchard in the Al-Salehia district, El-Sharkia Governorate (30°39′14″ N 31°52′28″ E and 30.653862° N 31.874371° E), this study was carried out on 12-year-old Naomi mango trees during the 2020–2021 seasons. The trees were planted in sandy soil at a distance of 2 m × 4 m (1250 trees/ha) with a drip irrigation system that used river Nile water. There were 2 JR hoses for each tree every 50 cm on the hose. There was a discharge point of 2 L per h. The Naomi mango cultivar was planted in 2010 after grafting onto ‘Succary’ rootstock.

### 2.2. Experimental Design

**Figure 1:**
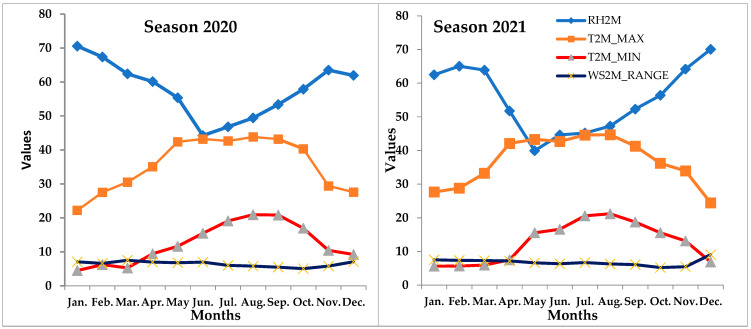
The average monthly of (T2M) temperature average at 2 m (°C); (TMIN) temperature at 2 m minimum (°C); (TMAX) temperature at 2 m maximum (°C); (WS2M) wind speed at 2 m during both growing seasons.

The application proceeded from November to March during the first season (2020/2021) and the second season (2021/2022). Three replications (9 trees per block) were created using the randomized block experimental design, with 4 treatments consisting of 2 different doses of nitric oxide (NO) 50 and 100 μM, fogger spray, and no treatment (control). The treatment of NO was applied 5 times (i.e., 1 November, 1 December, 1 January, 1 February, and 1 March) during the growing season, while fogger spray was used when temperatures fell below 4 °C from December to March. Fogging began at the same time as sprinkler irrigation before the temperature in the studied area was reduced. Utilizing a meteorological automatic station set up in the research area, temperatures were recorded as shown in the Figure 1. Each tree had a sprinkler along the line at the top, where fogging was administered to cover the full canopy of each tree. Every day when the temperature fell below 4 °C, fogging was applied for 5 min until the temperature reached 5 °C. The leaves of the experimental trees were uniformly covered in the NO substance, with 2 L of suspension sprayed per tree. To increase adherence to the leaf surface, all sprayed solutions, including the control (0%), contained 0.1% of tween 20 as a detergent. The location has a Mediterranean climate with average annual temperatures of 21.3 °C and rainfall totals of 26 mm, according to Rodrigues et al. [35]. A physical and chemical examination of the orchard soil surface (40 cm depth) was conducted prior to the start of the first season (2020), as indicated in Table 1 below. A total of 94.53% of the soil in the study region is sand (Table 1).

The recommended fertilization program was 300 N, 96 P_2_O_5_, and 240 K_2_O kg /ha/year for the Naomi mango tree in the region of the study. In March, May, and August, the micronutrient applied was a mixture of 300, 150, 100, 50, and 50 mg of the applied fertilizer made of chelated Fe, Mn, Zn, Cu, and B as boric acid, respectively.

### 2.3. Field and Laboratory Determinations

#### 2.3.1. Vegetative Growth

The number of damaged leaves per shoot was recorded. In this respect, 4 shoots in all origin directions around the tree canopy (i.e., north, east, south, west) and the total number of leaves per shoot were recorded in early March during both seasons. In April, a sample of 10 mature leaves per tree was abscised, and the leaf area (cm^2^) was estimated using Equation [36] as follows:

*Leaf area (cm^2^) = 0.70 (L × W) − 1.06* (1)

where: L and W = maximum leaf length and width, respectively.

#### 2.3.2. Flowering and Fruiting Data

The flowering date for each treated tree was recorded. The average number of panicles per tree (i.e., the average number of panicles in each replication) was also recorded. The number of flowers per panicle (i.e., 5 panicles from each replicate at full bloom) were chosen and the total number of flowers were counted and recorded at the full bloom phenological stage. The number of hermaphrodite (perfect) and male flowers per panicle were counted and recorded. Fruit number per tree were estimated from each treated time of harvest. The average weight of the fruit (g) was recorded as the average weight of 5 fruits from each replicate at harvest.

#### 2.3.3. Fruit Quality Data

Fruit weight was used to calculate yield per tree (kg). Harvesting was completed on 15 August in both seasons of the Naomi mango cultivar according to Khattab [37]. A yield (kg/tree) was recorded. The percentage was computed using the equation of Abd El-Naby [35].

(2) $$Yield\;increasing\;\left(\%\right)=-\frac{\left(Yield\;\left(treatment\right)-Yield\;\left(control\right)\times 100\right)}{Yield\;\left(control\right)}$$

Total soluble solids in fruit (TSS percentage) and total acidity percentage were determined. A digital refractometer was used to determine the TSS% of fruit juice (force–gauge model IGV-O.SA to FGV-100A, Shimpo instruments). Titration was used to quantify total acidity, which was expressed as citric acid according to AOAC [36].

#### 2.3.4. Photosynthetic Pigments

The chlorophyll content was assessed using a portable chlorophyll meter (SPAD502, Minolta, Japan) as the SPAD unit; these units were then converted to mg m^−2^ as specified by the following:

(3) $$Chlorophyll\;content\;\left(mg\;m^{-2}\right)=80.05+10.4\left(SPAD\;502\right)$$

where: SPAD 502 = chlorophyll meter reading (CMR).

#### 2.3.5. Determination of Proline Content

The rapid colorimetric method was followed [28] to estimate proline contents. Briefly, ninhydrin (1.25 g) was treated with glacial acetic acid (30 mL) followed by 6 M phosphoric acid (20 mL). The resulting mixture was heated until a clear solution was obtained and subsequently cooled and stored at 4 °C (stable for 24 h). Next, the explant material (0.5 g) was homogenized in 3% aqueous sulfosalicylic acid (10 mL) and subsequently filtered. The filtrate (2 mL) was treated with freshly prepared acid ninhydrin (2 mL) followed by glacial acetic acid (2 mL). After the resultant mixture was heated at 100 °C for 1 h, the reaction mixture was poured into ice. The obtained mixture was subsequently extracted with toluene (4 mL) and vigorously mixed for 15–20 s. The toluene phase was separated, and the absorption of the chromophore was assessed by spectrophotometer at a wave-length of 520 nm, utilizing toluene as a reference blank. The concentration of proline was finally evaluated from the standard curve and the fresh weight was defined.

#### 2.3.6. Electrolyte Leakage (EL) and Membrane Stability Index (MSI)

To assess membrane permeability, electrolyte leakage was calculated using a modified version of the method used by Guo et al. [26]. The youngest fully developed leaf of 10 randomly chosen plants was plucked from each replicate. The discs were then put in a 50 mL falcon tube and cleaned on the surface with distilled water 3 times. The discs were then incubated for 24 h at room temperature in a 50 mL falcon tube that contained 20 mL of deionized water (Aquinity2 P 10, MembraPure GmbH, Hennigsdorf, Germany). An electrical conductivity meter was used to measure the electrical conductivity of the bathing solution (EC1) following incubation (BALRAMA, Digital EC meter, New Delhi, India). The same samples were then heated in a water bath for 20 min, and after the solution cooled to room temperature, a second reading (EC2) was taken. The following formula was used to convert the EL into a % value:

(4) $$EL\;\left(\%\right)=\frac{EC1}{EC2}\times 100$$

Based on data on electrolyte leakage, the membrane stability index (MSI) was calculated and expressed as a percent value using the formula below:

(5) $$MSI\;\left(\%\right)=1-\frac{EC1}{EC2}\times 100$$

#### 2.3.7. Total Phenolic Compounds

The total phenolic content of Keitt mango tree leaves was calculated using the Folin–Ciocalteu colorimetric method and expressed as (mg/g) using gallic acid as a reference.

#### 2.3.8. Determination of Antioxidant Enzymes

Tissue preparation for enzymatic antioxidants: fresh leaf samples (0.2 g) were ground and homogenized in an ice bath in 4 mL homogenizing solution containing 50 mM potassium phosphate buffer and 1% (*w*/*v*) polyvinylpyrrolidone (pH 7.8). The homogenate was centrifuged at 14,000 rpm at 4 °C for 10 min and the resulting supernatant was used for enzyme assays. The absorbance was recorded on a spectrophotometer (Jenway 6305 UV/Visible) for 60 s.

##### Assay of Catalase (CAT)

The blank was prepared by mixing 0.05 mL of enzyme solution (supernatant) with 1.5 mL of 100 mM potassium phosphate classes buffer (pH = 7.2) and 0.5 mL of 75 mM hydrogen peroxide solution (H_2_O_2_). The distilled water was added to the mixture up to a volume of 3 mL. The reaction occurs by the addition of H_2_O_2_. The absorbance was recorded in a decrease at 240 nm for 60 s. The enzyme action was determined by calculating the quantity of decomposed H_2_O_2_.

##### Assay of Peroxidase (POD)

The determination of POD activity at 420 nm: The “BLANK” was prepared by mixing 0.05 mL of enzyme solution with 1.07 mL of 100 mM potassium phosphate buffer (pH = 6.0 at 25 °C), 0.3 mL of 5% (*w*/*v*) pyrogallol solution, 0.1 mL of 0.5% (*w*/*w*) H_2_O_2_, and 0.70 mL water.

##### Assay of Polyphenol Oxidase (PPO)

PPO activity was determined at 420 nm at 25 °C. The “BLANK” was prepared by mixing 0.05 mL of enzyme solution with 1.70 mL of 20 mM catechol solution (which was prepared in 50 mM potassium phosphate buffer, pH 6.8 at 25 °C).

(6) $$Enzyme\;activity\;\left(U/mL\right)=\frac{\left(\Delta Abs\times Vt\times 106\right)}{\left(\Delta t\times \varepsilon \times l\times Vs\times 1000\right)}$$

where ΔA is the change in absorbance, Δt is the time of incubation (min), ε is the extinction coefficient (M^−1^ cm^−1^), l is the cuvette diameter (1 cm), Vt is the total assay volume, and Vs is the enzyme sample volume (ml).

ε420 nm of pyrogallol is 2640 M^−1^.cm^−1^

ε420 nm of catechol is 2450 M^−1^.cm^−1^

ε240 nm of hydrogen peroxide is 43.6 M^−1^.cm^−1^

### 2.4. Statistical Analysis

The design of the present study was a complete randomized block design. The data obtained from the effects of the fogging system and nitric oxide on growth and yield traits of Naomi mango trees exposed to frost stress were statistically analyzed through a 1-way analysis of variance (ANOVA) using SPSS 21.0 software (IBM Corp, Armonk, NY, USA). Means of different treatments were compared using the Dunnett’s test (*p* ≤ 0.05) according to Snedecor and Cochran [38].

## 3. Results

### 3.1. Frost Adventurous and Leaf Physical Characteristics

**Figure 2:**
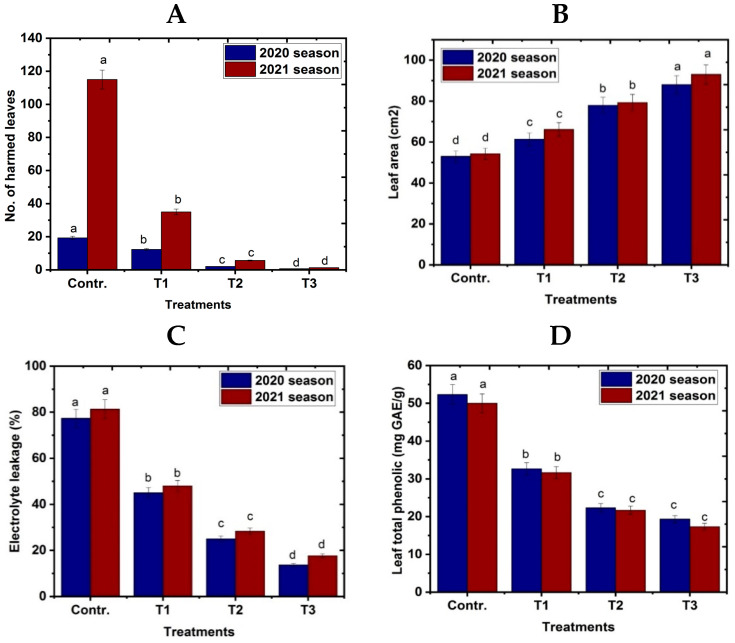
Effect of exogenous nitric oxide and fogger spray application on (**A**) no. of harmed leaves, (**B**) leaf area, (**C**) electrolyte leakage, and (**D**) membrane stability index of the Naomi mango cultivar under cold stress during seasons 2020 and 2021. Where T1 = NO at 50 μM; T2 = NO at 100 μM; T3 = fogger misting system. Bars indicate mean values ± SE (*n* = 9). Different letters above columns indicate significant differences among treatments at *p* ≤ 0.05.

The effects of spraying NO at various concentrations or using a fogger misting system on frost injury are depicted in Figure 2A,B. In comparison to the control treatment, the number of frost-damaged leaves per tree was reduced in all treatments using fogger misting systems and all spraying NO concentrations. The results showed that frost was definitely hazardous to shoots when the maximum proportion of injured leaves were observed on north shoots in the control treatment throughout the first and second seasons. However, as compared to the control treatment, the fogger misting system resulted in the lowest percentages of injured leaves in ascending order by NO treatment at 100 μM/L and 50 μM/L, respectively, for north shoots in all seasons. The vegetative development shows that foliar NO application and fogger spray treatments significantly enhanced leaf area in comparison to control treatments of mango in both seasons (Figure 2B). In this regard, fogger spray outperformed other evaluated treatments in descending order by spraying NO at 100 μM and 50 μM/L throughout the seasons when compared to the control. Data in Figure 2C,D showed that electrolyte leakage and the membrane stability index in Naomi mango trees grown under cold stress over 2020 and 2021 were significantly improved by foliar NO treatment and fogger misting systems. When compared to the control (stressed trees), the fogger misting system and foliar application of NO resulted in a decrease in electrolyte leakage and leaf total phenolic content of the tested cultivar. In contrast, adding NO and using a fogger misting system on the trees of the tested mango cultivar under cold stress increased the membrane stability index when compared to the control.

### 3.2. Leaf Chemical Characteristics

**Figure 3:**
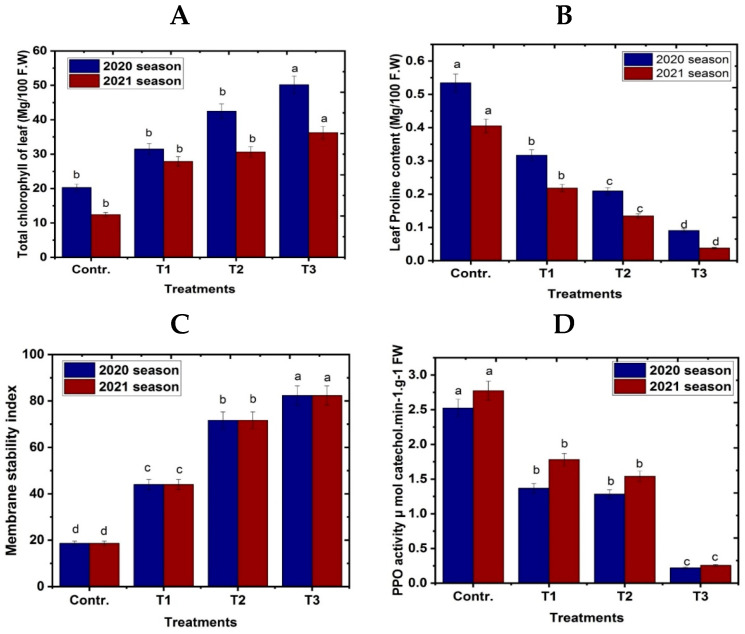

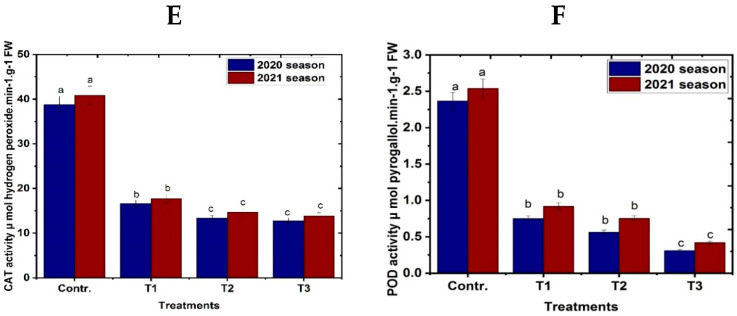
Effect of exogenous nitric oxide and fogger spray application on (**A**) leaf total chlorophyll content, (**B**) leaf proline content, (**C**) leaf total phenolic content and leaf antioxidant enzyme activity, (**D**) PPO, (**E**) CAT, and (**F**) POD of the Naomi mango cultivar under cold stress during seasons 2020 and 2021. Where T1 = NO at 50 μM; T2 = NO at 100 μM; T3 = fogger misting system. Bars indicate mean values ± SE (*n* = 9). Different letters above columns indicate significant differences among treatments at *p* ≤ 0.05.

Figure 3A shows how the content of leaf total chlorophyll content of Naomi mango trees changed during radiation frost with NO spray at various concentrations and a fogger misting system. When exposed to radiation frost, Naomi mango leaves treated with foliar sprays of various NO concentrations and fogger misting system treatments produced much more photosynthetic pigments than the control. In comparison to the control and other treatments, using a fogger misting system with a high dose of NO produced the greatest increases in total chlorophyll.

In addition, frost radiation stress significantly increased proline concentration and leaf total phenolic content in the leaves of Naomi mango trees compared with all other treatments Figure 3B,C. However, compared to the control (untreated), the addition of NO and the fogger misting system considerably reduced the concentration of proline. The fogger misting system’s lowest proline content was obtained when it was applied, as opposed to NO concentrations and untreated trees.

Results in Figure 3E,F illustrate that spraying nitric oxide at two levels and using the fogging spray system caused a decrease in mango leaf antioxidant enzyme activity, such as PPO, CAT, and POD, compared with control. The maximum antioxidant enzyme activity was achieved when the trees of the tested cultivar were exposed to frost stress conditions under the circumstances of the cultivated area (control stress untreated). On the other hand, the lowest value of antioxidant enzyme activity, such as PPO, CAT, and POD, were obtained following the application of the fogging spray system and spraying two concentrations of NO at 100 and 50 μM compared with control.

### 3.3. Flowering

**Figure 4:**
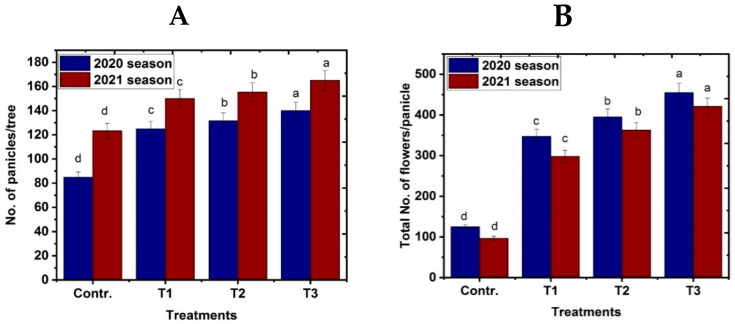
Effect of exogenous nitric oxide and fogger spray application on flower characteristics. (**A**) no. of panicles/tree and (**B**) total no. of flowers/panicle of the Naomi mango cultivar under cold stress during seasons 2020 and 2021. Where T1 = NO at 50 μM; T2 = NO at 100 μM; T3 = fogger misting system. Bars indicate mean values ± SE (*n* = 9). Different letters above columns indicate significant differences among treatments at *p* ≤ 0.05.

According to the results in Figure 4A,B, flowering in mango trees of the variety “Naomi” took place in the second half of March with both control and NO treatments, especially in the second season. Flowering occurs in the first season in the first half of March; however, the fogger misting system treatment causes early flowering in the second season during the second half of February or during the first half of March in the first season. Regarding the impact of nitric oxide spraying at different concentrations and the fogger misting system treatment on other flowering traits, such as the number of panicles per tree and the subsequent total number of flowers per panicle, the results obtained clearly demonstrate that control trees produced the lowest number of panicles per tree in comparison to other treatments. In the first and second seasons, the fogger misting system treatment increased the number of panicles per tree, followed in ascending order by NO at 100 and 50 μM/L, compared to other studied treatments. Consequently, there was a substantial difference between the different treatments and the control in the total number of flowers per panicle and the number of perfect (hermaphrodite) blooms.

### 3.4. Fruit Physical Characteristics at Harvest

**Figure 5:**
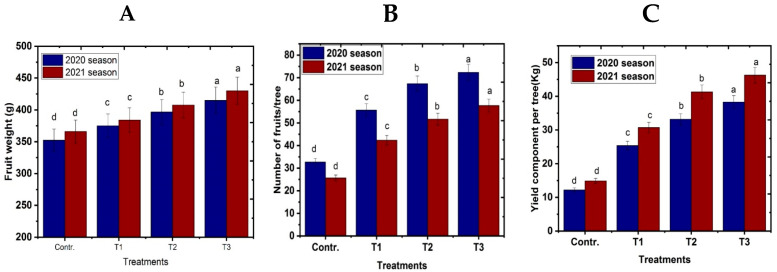
Effect of exogenous nitric oxide and fogger spray application on (**A**) fruit weight, (**B**) no. of fruits/tree, and (**C**) yield/tree of the Naomi mango cultivar under cold stress during seasons 2020 and 2021. Where T1 = NO at 50 μM; T2 = NO at 100 μM; T3 = fogger misting system. Bars indicate mean values ± SE (*n* = 9). Different letters above columns indicate significant differences among treatments at *p* ≤ 0.05.

Figure 5 made it abundantly clear that the fogger misting system treatment in the fall and winter months and spray of NO at concentrations of 50 and 100 μM/L on the Naomi cultivar foliage increased the number of fruits, fruit weight, and yield in comparison to the control in both seasons. The number of fruits per tree, fruit weight, and yield of the Naomi mango tree increased more with greater concentrations of NO spray at 100 μM/L and fogger misting system treatment than with other treatments or controls. The reduction in abiotic stress caused by NO and the fogger misting system may be responsible for the increase in fruit numbers, weight, and yield.

### 3.5. Fruit Chemical Characteristics at Harvest

**Figure 6:**
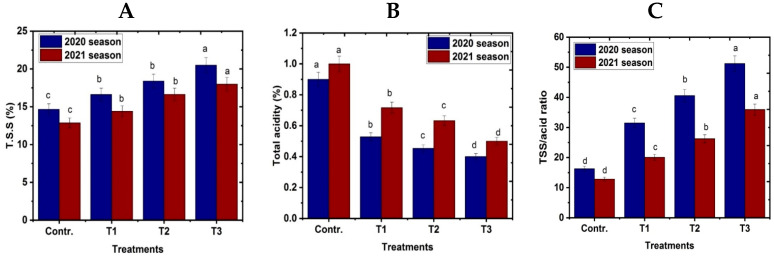
Effect of exogenous nitric oxide and fogger spray application on fruit chemical characteristics: (**A**) TSS%, (**B**) total acidity%, and (**C**) TSS/acid ratio of the Naomi mango cultivar under cold stress during seasons 2020 and 2021. Where T1 = NO at 50 μM; T2 = NO at 100 μM; T3 = fogger misting system. Bars indicate mean values ± SE (*n* = 9). Different letters above columns indicate significant differences among treatments at *p* ≤ 0.05.

Data in Figure 6A–C show that exogenous use of NO and the fogger misting system caused an increase in total soluble solids (TSS) and the TSS/acid ratio compared with the control. The fogger misting system possessed the highest values of TSS and TSS/acid ratio, followed in descending order by 100 and 50 μM of NO, respectively, compared with untreated tress. On the other hand, total acidity decreased with the application of NO and the fogger misting system compared with the control.

## 4. Discussion

**Figure 7:**
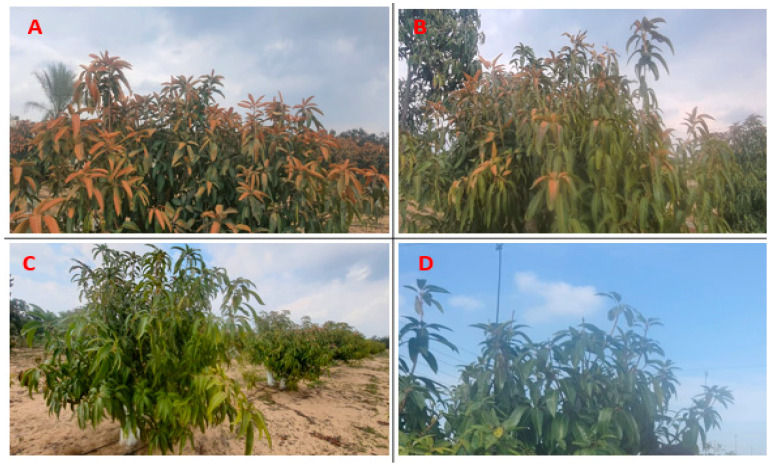
Effect of exogenous nitric oxide and fogger spray application on leaves of the Naomi mango cultivar under cold stress during seasons 2020 and 2021. (**A**) Control, (**B**) 50 μM of NO, (**C**) 100 μM of NO, and (**D**) fogger misting system.

Cold stress has a significant impact on a number of significant crop species, including paddy, rice, tomato, and several cucurbits [39,40]. It may result in leaves showing apparent symptoms, such as reduced size, withering, yellowing, and/or necrosis. Cold stress may cause lipid peroxidation and increase plant cell plasma membrane permeability [41]. Apart from phenotypic and physiological damage, cold stress imparting injuries at the cellular level can also be observed [42]. Cold stress causes dehydration, causing injuries to the plasma membrane [43]. Furthermore, due to comparatively less solute concentrations in apoplastic space, ice formation occurs, leading to dehydration [44]. Cold stress may also enhance the development of reactive oxygen species (ROS) in plants, leading to oxidative damage. When compared to the vast majority of systems now on the market, sprinkler irrigation can offer the highest level of protection [45]. It is also among the least expensive options for frost management. The amount of energy used by a sprinkler system is significantly less than what growers would typically spend on heaters and other electrical machinery [46]. Compared to these other approaches, it requires less labor and is comparatively less polluting [47]. Chlorosis, stunted seedlings, surface lesions on specific regions, curling leaves, discoloration, and tissue damage are the main morphological signs of cold stress [48]. Stem cracking, inadequate or nonexistent germination, a lack of vigor, metabolite leakage, leaf withering, necrosis, delay in regeneration, and inhibition of growth of vegetative propagated clones (such as alfalfa) are some of the problems that might occur [49]. If not handled properly, low temperatures below 0 °C and freezing conditions pose a serious threat to crop growth and could cause partial or complete losses [50]. Crop covers, fossil-fueled heaters, wind machines, foggers, and irrigation are all examples of active frost protection measures (alone or in combination with the other methods) [51]. Solid-set sprinkler irrigation is a cost-effective method to protect crops from frost damage [48]. Frost protection irrigation differs from normal irrigation in that water must be applied constantly and at high rates to the entire crop area throughout the frost event [47]. Even if ice forms on plant surfaces, the plant tissue temperature will not fall significantly below 320 °C, and frost damage will not occur [49]. Irrigation water generates heat, which is utilized to keep crops warm and prevent ice from developing inside plant tissue [50]. When the water is cooled, 8.4 BTU per gallon per degree Fahrenheit is emitted (sensible heat). When the water freezes from liquid to solid (latent heat), 1200 BTU are released per gallon. When a given volume of water freezes (from 0 °C water to 0 °C ice), it releases the same amount of heat as when it cools by 62.22 °C (1200/8.4), say from 80 to 0 °C [52]. Numerous studies have examined the effects of biotic and abiotic stress on apoplastic space [53]. Therefore, in the present study, the effects of exogenous NO on vegetative growth (leaf physical and chemical characteristics, flowering data, yield, fruit harvested data, and the activities of apoplastic SOD, CAT, and POX) were investigated in the leaves of the Naomi mango cultivar under cold stress conditions. Naomi mango leaf injuries decreased with exogenous NO treatments under cold stress (Figure 1 and Figure 7). As NO therapy before chilling may increase plant cell membrane mobility, it may lessen the percentage of leaf injuries [54]. The findings of the current study agree with those of earlier studies, such as that of Esim and Atici [55], who found the greatest results for freezing damage of maize from groups (plants without NO), while the average values of freezing leaves decreased by foliar application of NO at 100 μM. In addition, according to Chauhan et al. [56], foliar application of NO at concentrations of 150 and 300 µM significantly increased plant height, the number of leaves per plant, leaf area per plant, and leaf area index (LAI), while also showing a reduction in electrolyte leakage of chickpeas (*Cicer arietinum* L.) compared to the control at all stages. However, a lower concentration (150 µM) was more effective under cold stress. NO has been reported to help offset the effects of ROS in a variety of stressful situations. The findings of this study also suggest that NO and the fogger spray system contributed to Naomi mango tree cold resilience. As evidenced by the greater estimated MDA of trees treated with NO and the fogging spray system in both seasons, this study found that when trees were exposed to cold stress, oxidative damage from the excessive release of ROS may occur. Plants that experience cold stress struggle to grow and develop normally [57].

Due to cold stress, the growth of rice reproductive components during the flower opening stage was severely hampered, resulting in inflorescence sterility [58]. Additionally, cold stress reduces plant characteristics, such as germination, seedling vigor, growth, tillering, leaf withering, and pollen sterility, which results in lower agricultural yields [59]. The ice should be clear when the water on the plant has frozen, indicating that there was adequate water applied. If the ice is milky-white or hazy, the water application rate is insufficient to shield the blossom [60]. A frost event with calm, clear, radiative cold spells, little wind (0.5 ms^−1^), and high humidity (>90% by 0600 HR) would lessen the amount of heat lost by convection and evaporation and prevent the system from becoming ice-clogged. Sprinklers often freeze under these circumstances [46].

Over-crop (or over-canopy) sprinkling provides frost/freeze protection for both tall and short growing crops and for both types of frosts. Drawbacks include the very large amounts of water required, possible physical crop damage due to ice buildup, and problems with disease control, nitrogen leaching on sandy soils, and other soil water management issues due to the large volumes of applied water [61]. Water is delivered directly to the surface of the plant with over-crop sprinkling systems, where most of it might freeze and release heat (latent heat of fusion). However, if the wet-bulb temperature falls below the threshold temperature, this could cause catastrophic damage [62].

A gaseous phytohormone known as nitric oxide (NO) is engaged in a variety of physiological and developmental processes. It has been shown to improve antioxidant capacity, growth, chlorophyll, RWC, and photosynthesis. Additionally, it plays a crucial role in signaling and plant reactions in a variety of biotic and abiotic stressors [27]. In the present study, using NO at 50 or 100 μM led to an increase in chlorophyll content, as shown in Figure 2. These findings concur with those of other research where Fan et al. (2014) found that exogenous 100 μM of NO application somewhat increased the total chlorophyll content of Chinese cabbage seedlings under chilling stress compared with the control. This is consistent with earlier findings [41], where exogenous NO prevented the chlorophyll content from declining in response to chilling stress. Furthermore, Fan et al. [63] found that adding NO at 100 μM as a foliar application caused an increase in chlorophyll content of Bermuda Grass compared with untreated via NO. One of the trickiest problems is dealing with cold tension [64]. Chlorophyll-degrading enzymes (chlorophyllase) become more active at low temperatures and their biosynthesis is hindered, which causes the amount of chlorophyll in chilled plants to decrease [65]. Low chlorophyll levels in the leaves at cold temperatures can be interpreted as inefficient photosynthetic activity [66]. Additionally, the principal target of damage at low temperatures, PSII quantum efficiency, is correlated with a drop in photosynthetic capacity at those temperatures [67]. The major reason was that exogenous NO administration boosted antioxidant enzyme activity, which reduced chlorophyll degradation in seedlings caused by ROS. According to studies by Devacht et al. [68], and Sun et al. [69], cold temperatures decrease root metabolic activity, relative water content, and chlorophyll concentration.

Heat and cold stress are significant factors that limit crop output, along with other stresses. In plants, oxidative stress, lipid peroxidation, membrane damage, protein degradation, enzyme inactivation, color bleaching, and DNA strand disruption are brought on by high temperatures [70]. Similarly, cold stress induces several changes in biochemical and physiological processes, as well as ROS homoeostasis in plants [71]. In the current study, mango trees exposed to cold stress benefited from the foliar application of SNP up to 100 M after using NO and the fogging spray system in a number of developmental, physiological, and biochemical aspects. It is now well documented that osmolytes such as glycine betaine and proline improve enzyme and membrane integrity during cold stress [72]. It has been demonstrated that NO can elicit cold adaptation via proline synthesis in this setting [73]. In addition, some growers have implemented frost protection via over-tree microsprayer misting systems to reduce water requirements in cherry and peach trees under frost stress conditions [74].

NO was administered and a fogging spray was used, and the plant development significantly improved and the majority of the detrimental effects of cold stress were largely reversed. This eventually showed up in the yield. Nitric oxide (NO) has the potential to be used to increase the yield and quality of horticultural crop species since it controls plant development, promotes nutrient uptake, and activates disease and stress tolerance systems in the majority of plants. Even though NO research in model plant species is still in its early stages, it has already yielded a wealth of useful knowledge on horticultural crop species. New evidence shows how essential horticultural qualities can be provided by NO [71,72,73].

Our results support the viewpoint that NO and the fogger misting system caused an increase in TSS and the TSS/acid ratio compared with the control, as shown in Figure 4. Ren et al. [74] reported that adding NO led to an increase in total soluble solids while causing a reduction in total acidity in the fruits of the Tainong mango tree compared with the control. The low level of yield from the trees injured by winter frost was shown by Szymajda et al. [75]. A growing body of information is available on changes in S-nitrosated proteins after exposure to cold in horticultural crop species, including citrus, taking into account the significance of NO-dependent PTMs in NO signaling [76]. Future research should concentrate on the downstream signaling cascades that can be activated by NO-based S-nitrosation, as the majority of the proteins in cold-stressed plants that have been identified as S-nitrosated are involved in the metabolism. Similar to how plants under heat stress produce NO quickly, NO enhances heat tolerance by lowering ROS levels [77]. Crop damage from frost or severe heat has been demonstrated to be considerably reduced, or even completely prevented, by fog with water droplets of a specific size, according to research. This is due to the fact that the individual mist cloud droplets reflect the long-wavelength heat radiation that solid objects emit in these circumstances. In addition, the frost protection experiment was set up by spraying the fruit trees with water at different intervals for half a minute. The canopy of each tree contained four thermometers, the average value of which was used to characterize the efficiency of frost protection irrigation [78].

It has been reported that NO can regulate ROS metabolism through the modulation both of ROS-producing enzymes and of antioxidant systems [79]. NO is known to protect the plant against the oxidative stress which zucchini fruit undergo during cold storage, the resulting damage being higher in sensitive varieties such as Sinatra [80]. SOD, APX, GR, and CAT have been categorized as key enzymes for the defense against ROS for different types of stress and play an important role in the resistance of fruit to chilling injures (CI) [54,65]. NO can modify enzyme activity by protein *S*-nitrosylation, a specific and essential covalent posttranslational protein modification (PTM), by the incorporation of an NO group to a cysteine thiol [80]. NO also fortified cold tolerance in tomatos by regulating the expression of the transcription factor *LeCBF1* [81,82]. In particular, all enzymes of the ascorbate–glutathione (ASC-GSH) cycle appear to be controlled by NO through nitration and/or *S*-nitrosylation, with APX being the most widely studied enzyme involved in H_2_O_2_ detoxification [83]. Some reports suggest that all proteins from the AsA–GSH cycle are affected through *S*-nitrosylation and/or nitration through the interaction with NO [83]. Frost protection irrigation, while an effective strategy, plays a vital role in regulating this. Water application must begin in a positive temperature range [84], and water supply must be maintained continually below freezing. To avoid recoiling, frost protection irrigation must be maintained after the frost has passed. The most effective defense is to allow water to escape through the crown gap. In this scenario, a thin layer of water is applied to the entire surface of the tree, including the blooms. This is the most efficient method of frost protection for fruit trees. However, in some circumstances, particularly with lesser frosts, irrigation under the crown space might be an effective frost protection [85]. The key to protection with traditional over-plant sprinklers is to frequently reapply water at a rate high enough to prevent the temperature of the plant tissue from dropping too low between pulses of water. It is intended to constantly provide water at a lower application rate but specifically to a smaller surface area with non-rotating, focused over-plant sprinklers.

## 5. Conclusions

It can be concluded that spraying mango trees (i.e., four times) with NO and employing a fogging spray system can mitigate frost injury, improve blooming and the number of panicles per shoot, and increase the number of fruits per tree, fruit weight, and yield per tree. Furthermore, when trees were treated with NO at 100 μM, fruit quality, i.e., TSS content, TSS/acid ratio, low acidity content, and high values of leaf area, total chlorophyll content, and membrane stability index, were enhanced. However, the fogging spray application resulted in the highest values of any preceding parameter. On the other hand, using the NO fogging spray system caused a decrease in proline content, electrolyte leakage, leaf total phenolic content, and PPO, CAT, and POD antioxidant activity compared with the control, which indicated the beneficial effect of both of them in the current study.

## Figures and Tables

**Table 1:** Physical and chemical properties of the experimental farm soil.

Soil Physical Analysis						Soil Chemical Analysis							
Sand (%)	Silt (%)	Clay (%)	Soil Texture	EC (ds/m)	pH	Cations (meq/L)				Anions (meq/L)			
						Ca^++^	Mg^++^	Na^+^	K^+^	So4^−−^	CI^−^	HCo3^−^	Co3^−−^
93.53	4.22	2.25	Sand	0.46	7.20	2.00	0.94	1.24	0.21	0.48	1.87	2.00	0.00

## Data Availability

The data are presented in this article.
